# A Cysteine Protease Is Critical for *Babesia* spp. Transmission in *Haemaphysalis* Ticks

**DOI:** 10.1371/journal.ppat.1000062

**Published:** 2008-05-16

**Authors:** Naotoshi Tsuji, Takeharu Miyoshi, Badger Battsetseg, Tomohide Matsuo, Xuenan Xuan, Kozo Fujisaki

**Affiliations:** 1 Laboratory of Parasitic Diseases, National Institute of Animal Health, National Agriculture and Food Research Organization, Tsukuba, Ibaraki, Japan; 2 National Research Center for Protozoan Diseases, Obihiro University of Agriculture and Veterinary Medicine, Obihiro, Hokkaido, Japan; 3 Department of Infectious Diseases, Kyorin University School of Medicine, Mitaka, Tokyo, Japan; 4 Department of Emerging Infectious Diseases, School of Veterinary Medicine, Kagoshima University, Korimoto, Kagoshima, Japan; Stanford University, United States of America

## Abstract

Vector ticks possess a unique system that enables them to digest large amounts of host blood and to transmit various animal and human pathogens, suggesting the existence of evolutionally acquired proteolytic mechanisms. We report here the molecular and reverse genetic characterization of a multifunctional cysteine protease, longipain, from the babesial parasite vector tick *Haemaphysalis longicornis*. Longipain shares structural similarity with papain-family cysteine proteases obtained from invertebrates and vertebrates. Endogenous longipain was mainly expressed in the midgut epithelium and was specifically localized at lysosomal vacuoles and possibly released into the lumen. Its expression was up-regulated by host blood feeding. Enzymatic functional assays using *in vitro* and *in vivo* substrates revealed that longipain hydrolysis occurs over a broad range of pH and temperature. Haemoparasiticidal assays showed that longipain dose-dependently killed tick-borne *Babesia* parasites, and its babesiacidal effect occurred via specific adherence to the parasite membranes. Disruption of endogenous longipain by RNA interference revealed that longipain is involved in the digestion of the host blood meal. In addition, the knockdown ticks contained an increased number of parasites, suggesting that longipain exerts a killing effect against the midgut-stage *Babesia* parasites in ticks. Our results suggest that longipain is essential for tick survival, and may have a role in controlling the transmission of tick-transmittable *Babesia* parasites.

## Author Summary

Ticks are important ectoparasites among the blood-feeding arthropods and serve as vectors of many deadly diseases of humans and animals. Of tick-transmitted pathogens, *Babesia*, an intracellular haemoprotozoan parasite causing a malaria-like disease, called babesiosis, gain increasing interest due to its zoonotic significance. When vector ticks acquire the protozoa via blood-meals, they invade midgut and undergo several developmental stages prior to exit through salivary glands. It has long been conceived that midguts of these ticks evolve diverse innate immune mechanisms and perform blood digestion critical for tick survival. A cysteine proteinase, longipain, was identified from the three-host tick *Haemaphysalis longicornis*, which shows potent parasiticidal activity. Longipain is localized in midgut epithelium and its expression is induced by blood feeding. This protein is passively secreted into midgut lumen where it exerts enzymatic degradation of blood-meals. A series of experiments unveil that longipain-knockdown ticks when fed on *Babesia*-infected dog, exhibited a significantly increased numbers of parasites compared with controls. Longipain has shown to interact on the surface of *Babesia* parasites *in vitro* and *in vivo*, and is thought to mediate direct killing of the parasites, suggesting that longipain may be a potential chemotherapeutic target against babesiosis and ticks themselves.

## Introduction

The ixodid ticks are obligate hematophagous organisms that belong to the phylum Arthropda, and are classified with spiders and scorpions in the class Arachnida [1]. Ticks are long-term blood-pool feeders, while mosquitoes are short-term vessel-feeders, and the process of blood digestion in ticks differs mechanistically from that in haematophagous insects [2],[3]. After blood feeding, ticks can increase more than 50 times in body weight compared with their original weight due to the acquired host blood meal, which mainly consists of red blood cells. Blood digestion in ticks is a slow intracellular process that takes place via phagocytosis by desquamated epithelial cells in the midgut [4],[5]. The tick midgut is considered to contain evolutionally acquired molecules involved in host blood digestion [6],[7]. The existence of secreted proteolytic enzymes in blood-sucking ticks suggests that they are required for various functions necessary for survival via successful blood-feeding behavior, which includes continuous feeding for days, or even weeks [8],[9], However, the precise mechanism responsible for the host blood digestion is unknown.

Ticks as vectors are the most important ecto-parasites of domestic animals and are the second-most important vector next to mosquitoes among arthropods that transmit infectious diseases in human [10]. Pathogens, including viruses, bacteria and parasites, are taken up in the blood meal and exposed to a potentially hostile environment in the tick's midgut before invading the epithelium, where they subsequently multiply. Recent studies have shown that the midgut is involved in diverse arthropod innate immune responses against pathogens [11]–[14]. Thus, tick proteolytic enzymes in the midgut may play critical roles in host blood meal digestion and pathogen transmission. Such products may become increasingly important as drug targets and vaccine candidates for both tick control and tick-borne diseases [15]–[17].

The ixodid tick *Haemaphysalis longicornis* is an important disease vector for human and animal pathogens, including the causative agents of babesiosis, Q fever and Russian encephalitis. *H. longicornis* is the primary vector of the pathogens causing babesiosis of humans and domestic animals in Japan [18]. Babesiosis is a human malaria-like disease that has recently been considered as an emerging zoonosis [19]–[21]. Recent reports have shown that endo- and ecto-parasite cysteine proteases play numerous indispensable roles in the survival of the parasites [22]. We hypothesized that longipain, a tick cysteine protease, would play a specific physiological role in blood feeding and *Babesia* parasite transmission. We report here the characterization of longipain isolated from *H. longicornis*.

## Results

### A *longipain* cDNA

**Figure 1:**
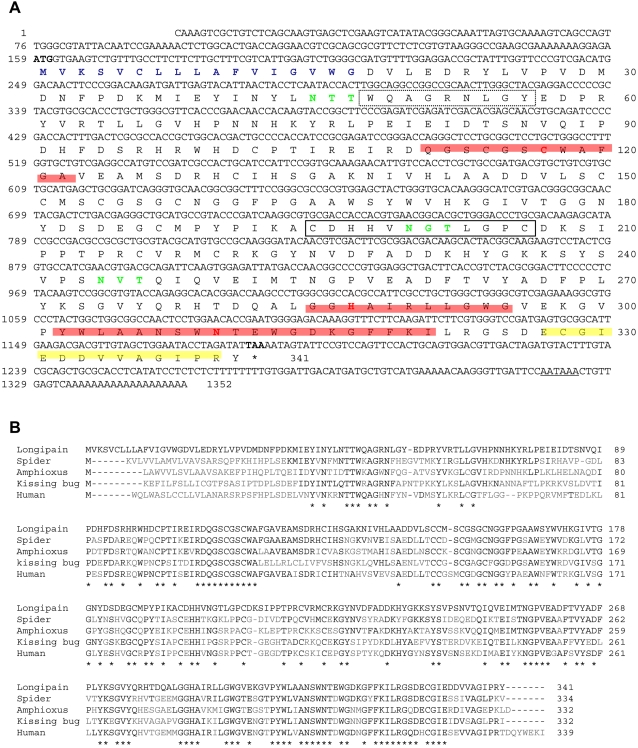
Characterization of *longipain* cDNA. (A) Nucleotide and predicted amino acid sequence of the longipain cDNA. The nucleotide sequence is numbered on the left and the deduced amino acid sequence is numbered on the right. The stop codon is indicated by an asterisk. Blue and green lettering indicate the signal peptide and *N*-glycosylation sites, respectively. Red highlighting indicates conserved motifs that form the catalytic triad of the active site in Clan CA cysteine proteases, including Cys-117 (red lettering), His-287 (red lettering), and Asp-307 (red lettering). Yellow highlighting marks the S_2_ pocket of cathepsin. The occluding loop of cathepsin B-like protease is shown boxed. The dotted box indicates the nine-amino-acid peptide loop suggesting mannose-6-phosphate-independent trafficking. (B) Alignment of the amino acid sequences of longipain and cathepsin B-like protein from *Araneus ventricosus* (GenBank accession number AAP59456), the amphioxus *Branchiostoma belcheri tsingtaunesehuman* (AAQ83887), the kissing bug, *Triatoma sordida* (AAT48984), and *Homo sapiens* (AAH10240). Identical residues are marked with an asterisk. Gaps, marked with hyphens, have been introduced for better alignment.

We isolated a longipain cDNA that was 1,352 bp long and included a start codon at nucleotides 159–161 with a consensus lower eukaryote initiation sequence (AXXATGG) and a stop codon at nucleotides 1182–1184. The ORF extending from position 159 to position 1184 codes for 341 amino acid residues. The 3′ untranslated region contained 149 bp and ended with an 18-bp poly (A)+ tail that began 10 bp downstream from AATAAA, which is the eukaryotic consensus polyadenylation signal. The predicted sequence showed the presence of a signal peptide of 17 amino acid residues, suggesting that longipain is secreted. The mature protein had a predicted molecular mass of 36,3335.2 Da and pI 4.33. Longipain belongs to the papain family of cysteine proteases [23] and possesses each of the conserved motifs identified in the active site of lysosomal cathepsins (Fig. 1A). Longipain also possesses an occluding loop motif that is thought to be responsible for the exopeptidase activity of cathepsin B-like proteases [23]. The nine amino acid peptide loop (residues 48–56) is suggestive of mannose-6-phosphate-independent trafficking [24]. The presence of three predicted *N*-glycosylation sites suggests that longipain may be a glycosylated protein. An NCBI search revealed that longipain possessed a conserved cathepsin L domain in addition to a cathepsin B domain. However, the conserved inter-space motif of cathepsin L that is structurally different from that of cathepsin B was not found in the pro-region of longipain [25]. The three-dimensional structures of vertebrate cathepsin B and L have revealed the important amino acid residues that contribute to the substrate specificity [26]. In these cathepsins, the substrate preference is primarily determined by the S2 subsite of the active site pockets [27]. The S2 pocket of longipain contains Asp332, like the human malaria parasite cathepsin L. Longipain was found to be most similar in sequence architecture to the known spider cathepsin B from *Araneus ventricosus*, with 64 % similarity (Fig. 1B). Longipain also shares similarity to some other GenBank™ sequences, including the cathepsin B-like protease of the amphioxus (*Branchiostoma belcheri tsingtaunesehuman*), the kissing bug (*Triatoma sordida*) and humans, to which the similarities were 57%, 56%, 55%, respectively.

### Characterization of endogenous longipain

**Figure 2:**
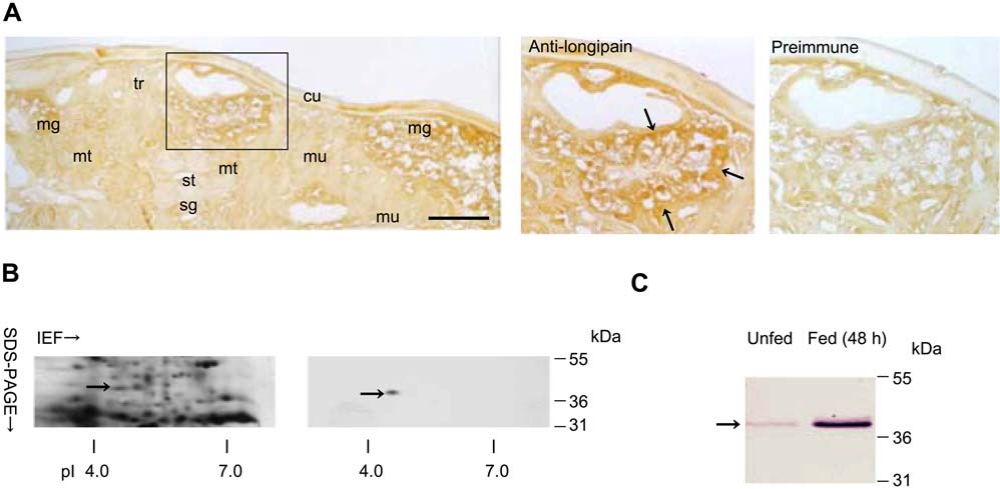
Endogenous expression of longipain. (A) Immunohistochemical localization of longipain. Flat sections of a whole partial-fed adult female tick were exposed to either mouse anti-longiapin antibody or pre-immune mouse serum. Areas marked by squares are shown at higher magnification in the right panels. cu, cuticle; mg, midgut; mt, malphigian tube; mu, muscle; ov, ovary; st, salivary duct; sg, salivary gland; tr, trachea. Arrows show endogenous longipain. (B) The molecular size of longipain. Fifty micrograms of tick-extract protein were separated by two-dimensional pH-gradient gel electrophoresis, and the proteins were then either transferred to a nitrocellulose membrane or stained with a 2D silver stain kit. The fact that endogenous longipain was bound to the anti-rlongipain serum was demonstrated by alignment between the stained gel and immunoblot membrane. The arrow shows endogenous longipain, which corresponded to the immunoreactive spot. (C) The level of longipain was increased by blood feeding. Whole tick extracts from the same number of individuals were subjected to immunoblot analysis. Soluble antigens from 3 individual nymphal ticks that were either unfed or partially fed (48 h after they started feeding) were analyzed by SDS-PAGE on a 10% acrylamide gel and transferred to a nitrocellulose membrane. The membrane was reacted with mouse anti-longipain serum at a dilution of 1∶800. The arrow shows endogenous longipain.

To identify the endogenous longipain in *H. longicornis*, we examined the localization of endogenous longipain by immunohistochemistry (Fig. 2A). The endogenous antigen bound to anti-longipain antibody was detected in club-shaped midgut epithelial cells protruding into the gut lumen of a partially fed adult [28],[29]. A positive reaction was not detected within the salivary gland or other tissues, suggesting that the endogenous longipain specifically expressed in the midgut. No positive reaction was detectable in either the internal or external tissues with preimmune mouse serum. Next, we performed two-dimensional immunoblot analysis (Fig. 2B). More than 200 visible protein spots appeared on silver-stained two-dimensional gels. Mouse longipain antibody strongly reacted with a protein of molecular mass 39 kDa and a pI of 4.5. To identify the internal amino acid residues, a protein spot was excised from extracts of adult female ticks using two-dimensional gel electrophoresis and processed as described previously [30]. After in-gel digestion with lysyl endopeptidase, peptides were collected by reverse phase high pressure liquid chromatography (RP-HPLC), and several peptides were analyzed to determine the internal amino acid sequence. The resultant sequences of the 39 kDa RP-HPLC-purified peaks were identical to those of the deduced amino acid sequence encoded by *longipain* cDNA. The sequencing confirmed that endogenous longipain has a molecular weight of 39 kDa with a pI of 4.5. To examine the expression pattern of longipain during blood-feeding, we evaluated the level of endogenous longipain in unfed and fed nymphs. Figure 2C shows that the expression of endogenous longipain was significantly increased after blood-sucking.

### Longipain is passively released into the lumen and localized in lysosomal vacuoles

**Figure 3:**
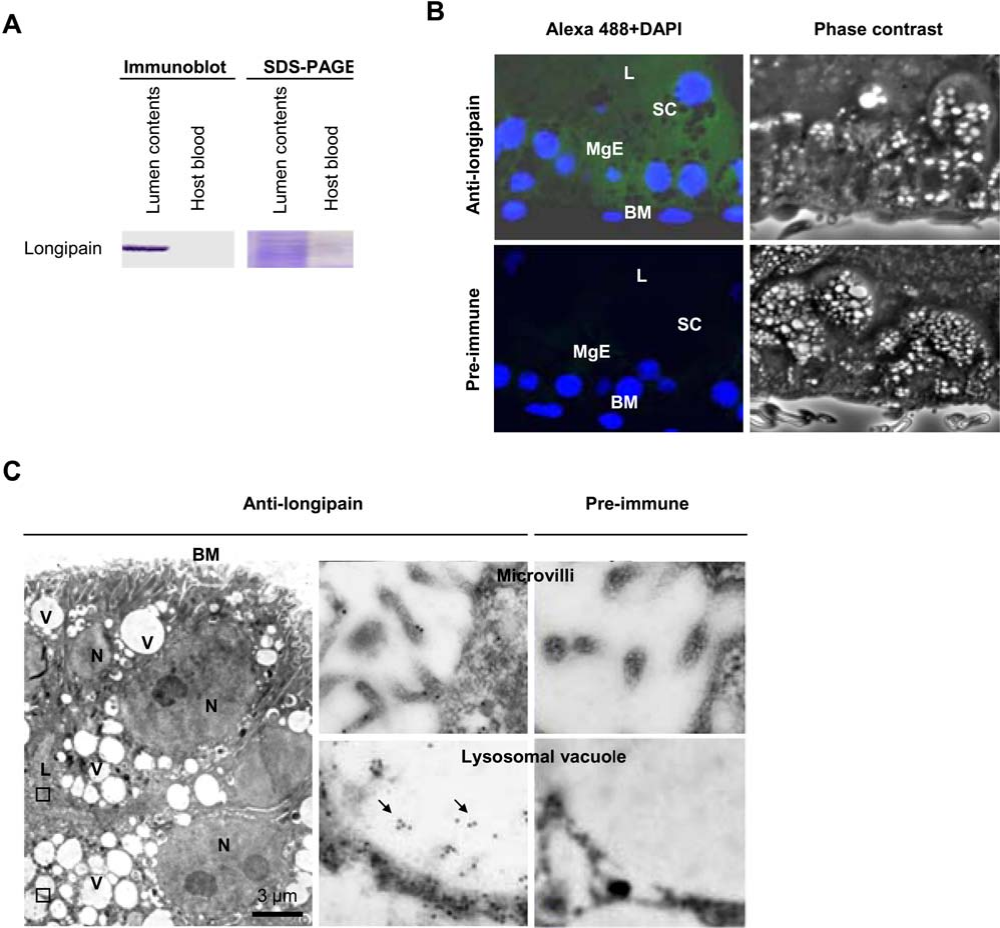
Subcellular and extracellular localization of longipain. (A) Longipain seen in the lumen contents of adult tick. The lumen contents were resolved by 8% SDS-PAGE and proteins were transferred to a nitrocellulose membrane and reacted with anti-longipain serum. (B) Longipain expressed in shedding cells. The midgut was analyzed by immunofluorescence microscopy at 48 h post blood-feeding. The endogenous longipian was visualized with anti-longipain serum and anti-mouse IgG Alexa 488 (green), and the nuclei of the midgut epithelial cells were stained with DAPI. BM, basement membrane; L, lumen; MgE, midgut epithelium; SC, shedding cell. (C) Ultrathin sections of the midgut epithelial cells. The sections were labeled with anti-longipain antibody followed by anti-mouse IgG coupled to 12-nm gold. Areas marked by squares are shown at higher magnification. Gold labeling of the midgut epithelial cells was found on the cell surface and in lysosomal vacuoles (Arrows). BM, basement membrane; V, vacuole; N, nucleus; L, lumen.

To determine the precise subcellular localization of longipain in the midgut, we performed immunoblotting, immunofluorescence and immunoelectron microscopy with anti-longipain. We were able to detect a positive band at 39 kDa in the lumen contents (Fig. 3A). Immunofluorescence analysis showed that endogenous longipain was expressed in the shedding cells (Fig. 3B). Prior studies suggested that shedding cells of the midgut are produced by passive release into the lumen [2],[31]. Furthermore, we detected endogenous longipain both on the surface and in the lysosomes of the midgut epithelial cells (Fig. 3C).

### Longipain possesses bimodal pH and temperature optima

**Figure 4:**
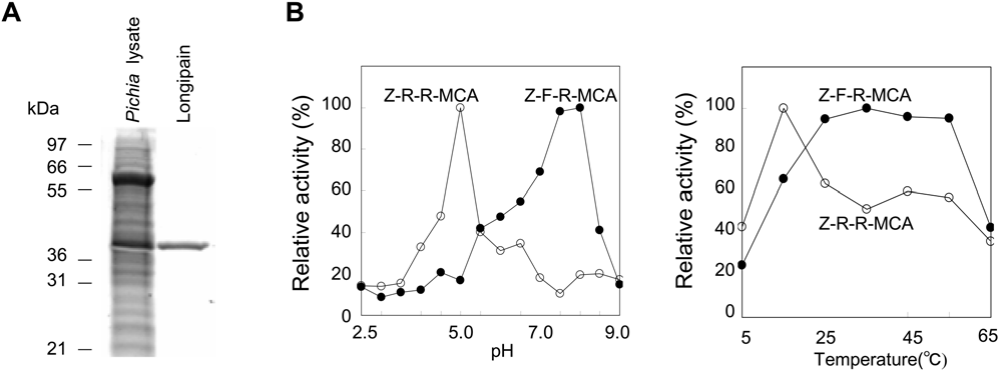
*In vitro* functional characterization of longipain. (A) Production of functional longipain. Longipain was expressed in *Pichia pastoris*. The purification process was monitored by 12.5% SDS-PAGE. (B) Effect of pH and temperature on longipain activity. Enzyme activity was assayed using Z-Arg-Arg-MCA (Z-R-R-MCA) or Z-Phe-Arg- MCA (Z-F-R-MCA) as the substrate as described in Experimental Procedures. The maximal proteolytic activity was taken as 100% and the relative activity was determined.

Mammalian cathepsin B and L are widely expressed cysteine proteases involved in both intracellular proteolysis and extracellular matrix remodeling [32],[33]. Recent reports demonstrated that parasite proteases function in a broader chemical environment than the homologous host environment [34]. We examined the hydrolysis activities using synthetic substrates to analyze the enzymatic function of yeast-expressed longipain (longipain, Fig. 4A). The activity assay using the purified longipain showed hydrolysis of Z-Arg-Arg-MCA and Z-Phe-Arg-MCA substrates (Table 1). The specific activity of longipain for Z-Arg-Arg-MCA was optimal at pH 5 and at temperature 15°C, and that for Z-Phe-Arg-MCA was optimal at pH 8 and at 35°C. In the assay of inhibition of cysteine protease activity, 10 µM E64 caused strong inhibition of longipain activity for the hydrolysis of Z-Phe-Arg-MCA (Table 2), but other inhibitors had little or no effect on the activity. E64 and PMSF had inhibitory effects on the ability of longipain to hydrolyze Z-Arg-Arg-MCA. Figure 4B shows the catalytic efficiencies of longipain from pH 3 to 9. The peak of activity with cathepsin B as substrate occurred at pH 3.6. Hydrolysis of cathepsin L as substrate showed dramatically different features, exhibiting a broad peak of catalytic efficiency from pH 7 to 8. Next, we examined the temperature dependency of longipain activity and found that the catalytic efficiency also showed different optimal temperatures between the two substrates. Longipain had good activity against cathepsin B at 15°C, while cathepsin L was strongly preferred at 37°C. Our results demonstrate that longipain functions not only over a broad range of pH conditions, but also over a wide range of temperature conditions.

**Table 1:** Hydrolysis of fluorogenic substrates by longipain

Substrate^a^	*K*_m_ (µM)	*k*_cat_ (s^−1^)	*k*_cat_/*K*_m_ (µM^−1^ s^−1^)
Z-Arg-Arg-MCA (substrate for cathepsin B)	16.0±0.2	8.0×10^2^	50
Z-Phe-Arg-MCA (substrate for cathepsin B/L)	12.1±0.08	5.5×10^2^	45
Suc-Leu-Leu-Val-Tyr-MCA (substrate for chymotrypsin)	NH^b^		

a Five micrograms of longipain (5 µg) were included in the standard reaction mixture containing 25 mM citric acid/sodium phosphate, pH 6.0, 5 mM DTT, 0.4 mM substrate in a final volume of 100 µl. The hydrolytic activity was monitored by spectrofluorometry at 360 nm for excitation and 465 nm for emission.

b NH, not hydrolysed.

### Longipain cleaves spectrin, a major component of the erythrocyte membrane

**Figure 5:**
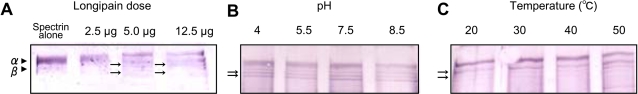
Hydrolysis of rabbit erythrocyte spectrin by longipain. Ten micrograms of native spectrin were incubated with longipain for 6 h. The hydrolysis pattern was analyzed by 6% SDS-PAGE and proteins were transferred to a nitrocellulose membrane and reacted with rabbit anti-human spectrin serum. (A) Dose-dependant hydrolysis of rabbit erythrocyte spectrin. Spectrin alpha and beta are indicated by arrowheads. The hydrolytic fragments are indicated by arrows. (B) Effect of pH on the hydrolysis of spectrin. (C) Effect of temperature on the hydrolysis of spectrin. The results demonstrated that longipain hydrolyzed rabbit erythrocyte spectrin over a wide range of pH and temperature.

The contents of the blood meal in the midgut are composed of a variety of native proteins derived from the hosts. To identify the endogenous substrates in the midgut, several components of host blood were tested for hydrolysis by rlongipain. Although a proteolytic effect was not shown against most of the components, longipain was shown to successfully cleave spectrin, a major component of erythrocyte membranes. Erythrocyte spectrin is a heterodimer composed of a 280-kDa α subunit and a 246-kDa β subunit which associate in a side-to-side, antiparallel configuration to form a 100-nm rod-like structure. Spectrin in other tissues may be composed of distinct but homologous α and β subunits, and is sometimes referred to as fodrin [35]. Figure 5 shows the hydrolysis of host erythrocyte membrane components by longipain. Spectrin α and β subunits reacted strongly with anti-human spectrin antibody (arrowheads). Fragments with smaller molecular mass, corresponding to hydrolyzed spectrin, were detected below the intact form on the immunoblot. The results obtained with various amounts of longipain indicated that the hydrolysis of spectrin seemed to be a dose-dependent reaction. The longipain showed strong hydrolysis of spectrin at pH 4.0–8.5, 20–50°C. Ticks can feed only on blood to obtain nutrients. Thus, spectrin may not be the only endogenous substrate for longipain in the tick midgut. Hemoglobin was used as a substrate and was not hydrolyzed by longipain.

### Longipain kills *Babesia* parasites

**Figure 6:**
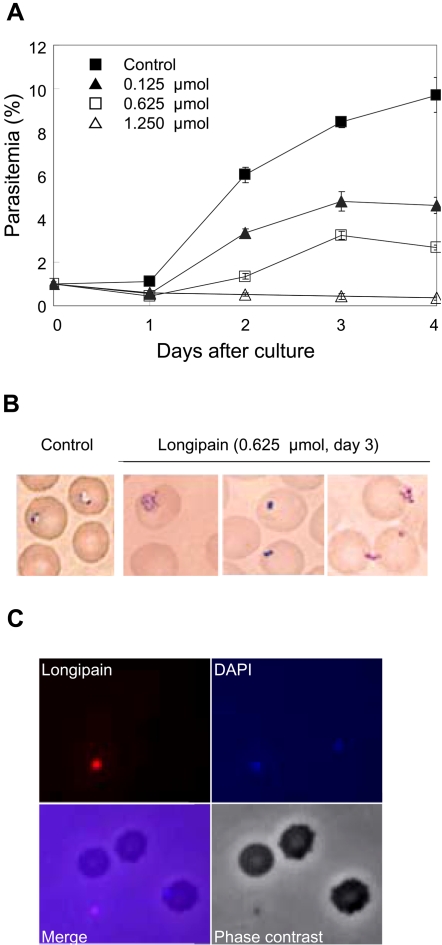
Longipain directly inactivates *Babesia* parasites. (A) Longipain kills *Babesia* parasites. Longipain was incubated with *B. equi-*infected erythrocytes (1% parasitemia) in culture medium. Parasite-infected erythrocytes were counted as the percentage of total erythrocytes. (B) Representative images of the parasites treated with longipain. Longipain blocked parasite invasion but not host erythrocyte rupture. (C) Longipain binds to the surface of *B. equi* merozoites. Biotin-labeled longipain was visualized with anti-longipain serum and Alexa 594 streptavidin (red), and the nuclei of parasites were stained with DAPI. The images suggest that the babesiacidal effect occurs via specific adherence to the parasite membrane.

Prior reports suggested that vector proteases facilitate the invasion of pathogens [36],[37]. It was therefore of interest to test the hypothesis that longipain would enhance the spread of *H. longicornis*-bearing *Babesia* parasites after their release from the infected erythrocytes. We incubated the equine *Babesia* parasite *B. equi* in medium supplemented with longipain. Longipain inhibited merozoite proliferation at a concentration of 0.125 µmol (Fig. 6A). The inhibition was dose dependent. Giemsa-stained smears of the culture medium showed abnormal multidividing forms and pyknosis of *B. equi* parasites (Fig. 6B). Free merozoites were also found in the culture medium. No morphological changes of erythrocytes were seen in the presence of longipain at any concentration. This finding suggests that longipain may react with intraerythrocytic substrates rather than membrane-bound components. Next, *B. equi* were incubated with biotin-labeled longipain in culture medium to explore how longipain interacted with the parasites. Fluorescence microscopy showed positive reactions at the surface of the free-merozoites, but not erythrocytes (Fig. 6C). We therefore assume that the killing of *Babesia* parasites in the midgut of *H. longicornis* tick is meditated by several midgut-derived proteolytic enzymes.

### RNAi-treated ticks have decreased feeding capacity and increased parasite transmission ability

**Figure 7:**
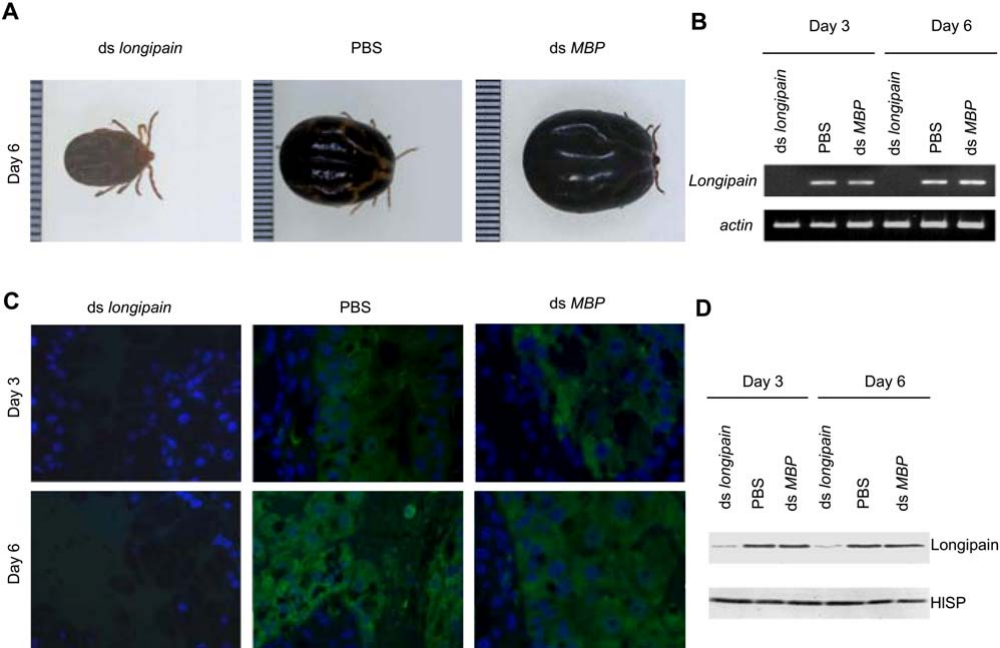
Gene silencing of *longipain* expression by RNA interference. Unfed adult female ticks were injected with *longipain* dsRNA into the haemocoel through the fourth coxa using fine-point glass needles. Control ticks were injected with PBS alone or *MBP* dsRNA. Ticks were collected from the ear of a *Babesia gibsoni*-infected dog on day 6 after attachment. One of three representative RNAi experiments is shown. (A) Engorged ticks at day 6. The body weight of ticks with silencing of *longipain* was significantly lower than that of the control ticks. Pre-oviposition, oviposition and egg periods of ticks treated with dsRNA were similar to those of PBS-control ticks. One small unit of the scale equals 0.5 mm. (B) RT-PCR analysis. Note the reduced expression of *longipain* mRNA in *longipain* dsRNA-injected ticks. (C) Injection of *longipain* dsRNA inhibited endogenous longipain expression (green) in the midgut (×600). (D) Immunoblot analysis of endogenous longipain expression. The results show the absence of longipain expression, demonstrating that effective knockdown of *longipain* mRNA was achieved by dsRNA treatment. HlSP; *H. longicornis* serine protease [38].

**Figure 8:**
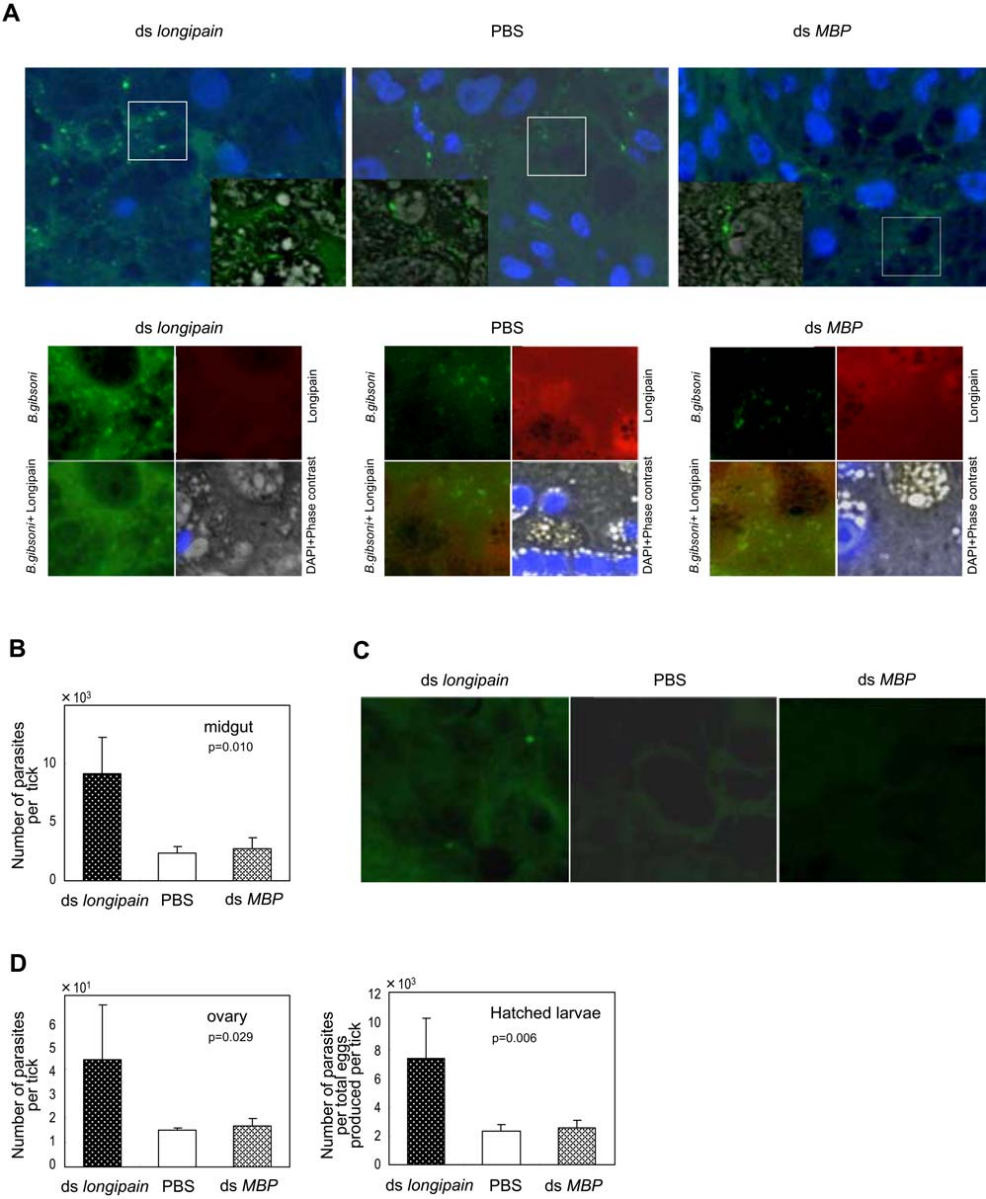
Knockdown of *longipain* by RNA interference facilitates transmission of *Babesia* parasites through the vector ticks. The vectorial capacity of ds *longipain* RNA-treated ticks was evaluated by immunofluorescence microscopy and quantitative-PCR analysis. (A) Distribution of *B. gibsoni* and endogenous longipain in the midgut at 6 days of feeding. Upper panels: *B. gibsoni* were visualized using mouse anti-*B. gibsoni* antibody (green), and the nuclei of the midgut epithelial cells were stained with DAPI (blue). Areas marked by squares are shown as merged images consisting of immunofluorescence and phase contrast images at higher magnification in the corner of the original panel. Lower panels: *B. gibsoni* and endogenous longipain in the lumen. *B. gibsoni* were visualized using mouse anti-*B. gibsoni* antibody (green) and rabbit anti-longipain (red). Note the increased number of parasites in the lumen and the midgut epithelial cells of *longipain* dsRNA-injected ticks. (B) Prevalence and severity of *B. gibsoni* infection at day 6. The numbers of parasites invading the midgut epithelial cells were evaluated by measuring the P18 gene in the *B. gibsoni* genome DNA using real-time quantitative PCR. The genomic DNA was extracted from the midgut of which the lumen contents had been removed. The bars indicate means and the error bars indicate s.e.m. for three independent experiments of five ticks. (C) Representative image of migrated *Babesia* parasites in the ovary (×800). Parasites were detectable in the 50 serial sections of the *longipain* dsRNA-injected ticks but not in those from the PBS group. (D) Level of *B. gibsoni* infection in the ovary and eggs. The quantitative results demonstrated that repression of *longipain* enhanced the *B. gibsoni* infection in the vector tick. The bars indicate means and the error bars indicates s.e.m. for three independent experiments of four ticks.

We hypothesized that endogenous longipain may promote host blood digestion and at the same time decrease *Babesi*a parasite survival in *H. longicornis*. To test these possibilities, we used RNA interference (RNAi) to knockdown *longipain* mRNA by dsRNA in adult *H. longicornis*. RNAi, a phenomenon in which double-strand RNA (dsRNA) silences gene expression through specific degradation of the cognate mRNA, is a direct and efficient way of producing and identifying the loss-of-function of targeted genes as a reverse genetic tool. In this study, the dsRNA-treated ticks were attached to a dog preinfected with the canine *Babesia* parasite *B. gibsoni*. We then assessed whether the transmission of *Babesia* parasites was affected by the endogenous longipain. Ticks injected with phosphate buffered saline (PBS) alone or with dsRNA generated from *E. coli* MalE maltose-binding protein (MBP) gene were used as control. Although *longipain* dsRNA-treated (mean±standard deviation; 84.0±10.9 mg, n = 6), PBS-treated and *MBP* dsRNA-treated ticks (PBS; 85.2±14.7 mg, n = 6, MBP; 86.2±12.4, n = 6) showed similar behavior of blood-feeding by day 3, longipain depression clearly impaired tick blood feeding after day 4. Ticks feed rapidly on a host before engorgement [2] although the underlying mechanism responsible for the developmental effect is unclear. The engorged adult ticks in the *longipain* dsRNA-treated group had a smaller and rounder appearance than those in the PBS-treated and *MBP* dsRNA-treated groups, and none had cuticular wrinkles on the dorsum, as were found in ticks in control groups (Fig. 7A). Significant differences in body-weight at engorgement were observed between the knockdown (mean ±standard deviation; 108.5±34.1 mg, n = 10) and control groups (PBS; 322.1±49.8 mg, n = 16: MBP; 322.9±44.0, n = 10). Reverse transcription polymerase chain reaction (RT-PCR) analysis revealed that injection of longipain dsRNA caused complete loss of longipain mRNA (Fig. 7B). This indicates that the significant reduction in longipain mRNA expression was due to a gene-specific dsRNAi effect. A decrease of endogenous longipain was also seen by immunofluorescence and immunoblot analyses (Fig. 7C, D). The midguts of *longipain* dsRNA-treated, PBS-treated and *MBP* dsRNA-treated ticks expressed almost equal amounts of *H. longicornis* serine protease (HlSP, data not shown). HlSP was previously identified from the midgut of *H. longicornis* and was shown to be associated with host blood feeding and digestion by our reverse genetic studies [38]. The present results strongly indicate that longipain dsRNA was efficiently delivered to the midgut of ticks via injection at the fourth coxae. *H. longicornis* is a three-host tick whose larvae, nymph, and adults all engorge on animals [18]. Thus, *H. longicornis* can transmit *Babesia* parasites interstadially through both larva to nymph and nymph to adult transition. *Babesia* parasites in the midgut lumen of the adult *H. longicornis* after blood feeding invade the epithelium, move to the ovary and finally arrive at the eggs [19]. We assessed whether *Babesia* parasites were affected by the suppression of endogenous longipain. Immunoflurorescence analysis revealed an increase in the number of *B. gibsoni* in the midgut lumen and in the epithelium of *longipain* dsRNA-treated ticks as compared to those of PBS-injected and *MBP* dsRNA-treated ticks on day 6 after injection (Fig. 8 A, upper panel). We then examined the localization of endogenous longipain and *B. gibosoni* using anti-longipain and anti-*B. gibosoni* antibodies by double immunostaining, in order to obtain more visible interaction between longipain and *Babesia* parasies. Double-immunostaining data clearly revealed the complete absence of longipain-mediated endogenous interaction in the midgut microenvironment, showing only an increased number of *B. gibsoni* in the longipain-knokdown ticks compared to those served as PBS-injected and MBP-treated controls (Fig. 8A, lower panel). This evidence suggests that longipain exerts its effect directly on the *B. gibsoni* parasites and mediates killing of the parasites. Actually, quantitative PCR analysis clearly demonstrated increased number of *B. gibsoni* in the midgut, consistent with the results of immunofluorecence staining (Fig. 8 B). In the ovary, *B. gibsoni* was detected in longipain-knockdown ticks, but not in control ticks (Fig. 8 C). The *longipain* dsRNA-treated ticks showed a significant increase in the number of *B. gibsoni* in the ovary and the hatched larvae as compared to those of PBS-injected and *MBP* dsRNA-trated ticks by quantitative PCR analysis (Fig. 8 D). Longipain-knockdown ticks showed approximately a 3-fold increase in the ability to transmit *Babesia* parasites. Together, these results confirm that longipain-mediated killing may regulate the number of *Babesia* parasites in the tick midgut.

## Discussion

Tick-borne disease is a major public health issue in many parts of the world, where the increasing prevalence of drug resistance underscores the need to identify new drug targets [39],[40]. Examination of metabolic pathways such as those involved in digesting the host blood meal has provided numerous attractive candidates for chemotherapeutic development, since blood-feeding is essential for tick survival [41],[42]. The molecular basis of how the digestion is maintained in nature during the complex life cycle of ticks is poorly understood. We speculate that ticks possess specific gene-products that have been acquired during the process of their evolution for host blood feeding. Our results obtained in the present study demonstrate that longipain is a multifunctional cysteine protease that functions in host blood meal digestion and in the regulation of the vectorial capacity for tick-borne *Babesia* parasites.

We confirmed here that longipain is passively secreted into the lumen of the midgut. Thus, it is likely that the process of digestion of the host blood meal occurs in the midgut lumen [43]. As the results of histological studies showed that longipain may function in two different physiological locations (the lysosomes of the epithelium and the midgut lumen), we concentrated our subsequent enzymatic studies on longipain. *In vitro* enzymatic functional assays revealed distinct pH and temperature preferences of longipain for the activity against cathepsin B and L substrates, indicating that longipain may be involved in anatomically specific activity in the midgut. In the present study, longipain was found to be released into the lumen and localized in lysosomes, suggesting that the activity in these locations is dependent on the pH conditions in the lumen and in the epithelium. Interestingly, the optimal temperatures for the synthetic cathepsin B and L substrates were also distinct. These results prompted us to examine whether longipain possesses a strong ability to cleave endogenous substrates derived from the host blood meal. Furthermore, we hypothesized that major protein components of the blood meal are targeted for hydrolysis over a wide range of pHs and temperatures. Interestingly, we were able to detect cleaved forms of spectrin upon hydrolysis by longipain. The developmental cycle of ticks involves two distinct drastic patterns [1]: a blood-feeding phase that occurs upon attachment to the host and a non-invasive phase that occurs during the period from engorgement until a subsequent attachment to the next host. The ambient temperature in vector ticks varies from the host body temperature to lower temperatures. The broad enzymatic properties of longipain might be related to the behavioral features of blood-feeding ecto-parasite ticks, i.e., whether feed on the host or remain off the host. Longipain functions as a protease virtually both at acidic and at neutral pH, in addition to functioning over a wide range of temperatures. Longipain may substitute for the functions of cathepsin B and L in ticks [44]. Taken together, our results using *in vitro* and *in vivo* substrates strongly suggest that longipain may have an expanded role beyond host blood digestion, as demonstrated by its pH preference and location in the midgut. Longipain may function in a broader range of environments in order to promote the survival of ticks.

The ixodid ticks have four developmental stages (egg, larva, nymph and adult) in their life cycle. Prior studies showed that the processes of digestion of the host blood meal in the midgut are distinct at different developmental stages of *H. longicornis* [2],[29]; pinocytosis occurs only at the nymph stages, while both phagocytosis and pinocytosis occur at the adult stage. It is likely that endogenous longipain may function stage-dependently against host blood components in the tick midgut. Ticks must acquire nutrients from the host blood meal and metabolize these nutrients via catabolism and anabolism. The unique life cycle and resulting microenvironment of ticks has led to the evolution of metabolic pathways which differ from those in mammalian hosts. An enzyme cascade of proteolysis of host blood components has been elucidated in blood-feeding helminths, hookworms and schistosomes, and human malaria parasites [45]–[48]. Our group has identified various proteases involved in host blood digestion from the midgut of *H. longicornis* [38],[41]. Intriguingly, to digest the meal, the unique haematophagus physiology of ticks relies predominantly on proteases that are distinct from those used by insects as well as from those in endo-parasites [2],[49]. Elucidation of the molecular mechanisms by which midgut proteases digest the host blood meal may make it possible to exploit the unique pathways and enzymes in the design of control strategies.

The interaction between ticks and pathogens in the midgut constitutes a critical aspect of disease transmission and a potential target for efforts to control tick-borne diseases [50],[51]. At the vector stage in *H. longicornis*, *Babesia* parasites must complete a complex developmental cycle in the tick in order for transmission to occur. After the release of *Babesia* via erythrocyte rupture, the parasites must invade into the epithelial cells of the midgut. Their development depends on the balance between the ability of the tick to establish a defense response against the parasite and the ability of the parasite to escape the tick's immune response [52]. Proteolytic enzymes from the midgut of ticks may inhibit the proliferation of the midgut-stage parasites. We hypothesized that *H. longicornis* possesses a specific gene product that exerts a partial protective response against *Babesia* in the midgut. Our results *in vitro* demonstrated that longipain kills the merozoite stage parasites released from erythrocytes in the case of equine *Babesia* parasites. Intriguingly, a cysteine protease purified from the venomous protein in the midgut of the social aphid exerts a killing effect against enemies, and its insecticidal activity may play a role in colony defense [53]. This biological function is suggestive of the evolutionary route of the aphid-specific molecules. *H. longicornis* might have evolutionally acquired babesiacidal activity in relation to becoming a vector of *Babesia* parasites. The killing effect points to a possible link with the structural features of longipain, which remains to be explored in the future.

Our reverse genetic analysis revealed that longipain regulates the blood meal digestion, demonstrating that loss of function causes a macroscopically detectable delay of blood-sucking speed, followed by a gain of body weight, suggesting that the effect of *longipain* dsRNA is specific to the protein being targeted. Longipain might function as a proteolytic enzyme in the process of blood-meal digestion in the lumen and the epithelium. Prior studies showed that a disease-bearing vector transmits only a limited number of parasites during blood-feeding, suggesting the existence of a partially successful natural defense mechanism against the parasite [54]. In the malaria vector (the mosquito), midgut serine protease is involved in regulating the parasite burden and the ability of *Plasmodium* parasites to invade into the midgut epithelium [55],[56]. An excessive number of parasites might destroy the midgut epithelium, resulting in the haemolymph flowing into the lumen, causing the death of the tick. A decreased level of proteolytic enzymes in the midgut of disease vectors might have a major impact on vectors and pathogens. Thus, we hypothesize that longipain acts as a defense molecule against invading *Babesia* parasites in *H. longicornis*. We found that longipain was highly expressed in the midgut, where it was localized in extracellular and intracellular parts of the epithelium. Knockdown of longipain induced a significant increase in the number of parasites in the lumen, suggesting that longipain directly kills *Babesia* parasites in the midgut. The recruitment of similar receptor and ligand interactions in both vector ticks and mammals in the fight against infection suggests that they have developed similar mechanisms and molecular pathways to recognize and eliminate the invaders [14],[57],[58]. These longipain knockdown studies revealed a substantial reduction in the proportion of host blood feeding and a corresponding increase in the proportion of the number of parasites in the midgut as well as the organs subsequently infected during the parasite life cycle. The fact that longipain is involved in the killing of the dog *Babesia* parasites suggests the existence of cross-talk between the tick immune response of *H. longicornis* and *Babesia* parasites. It is likely that diverse arthropod innate immune responses against *Babesia* parasites may be conserved and may contribute to controlling the tick vectorial capacity [59],[60].

In summary, we have identified longipain, a multifunctional cysteine protease from a babesial parasite vector tick. The present findings strongly suggest that longipain plays dual roles in the host blood meal digestion and in the control of transmission of the *Babesia* parasite. Our data demonstrate the pivotal role of longipain in the maintenance of the babesial vector ticks. Understanding the function of the midgut molecules that participate in the interaction between the *Babesia* parasite and vector ticks will lead to novel approaches to the control of animal and human babesiosis and provide a model for tick-borne diseases.

## Materials and Methods

### Ticks

*Haemaphysalis longicornis* (Okayama strain) were maintained at the Laboratory of Parasitic Diseases, National Institute of Animal Health, Tsukuba, Ibaraki, Japan, on rabbits as described previously [31].

### *Babesia* parasites

The *Babesia* parasites used in this study were as follows: a horse *Babesia* parasite, *Babesia equ*i, and a dog *Babesia* parasite, *B. gibsoni*. The U.S. Department of Agriculture strain of *B. equi* was maintained by *in vitro* culture at the National Research Center for Protozoan Diseases (NRCPD) [61]. The NRCPD strain of *B. gibsoni* was maintained in chronically infected dogs at NRCPD [62].

### Animals

All animals used in this study were acclimatized for 2 weeks prior to experiments. Animal experiments performed at the National Institute of Animal Health (NIAH) were conducted in accordance with the protocols approved by the NIAH Animal Care and Use Committee (Approval nos. 441, 508, 578). Animal experiments carried out at Obihiro University of Agriculture and Veterinary Medicine (OUAVM) were conducted in accordance with the Guiding Principles for the Care and Use of Research Animals promulgated by OUAVM (Approval nos. 6–42, C-2).

### Construction of tick cDNA library

Prior studies have shown that animals can be rendered immune to tick infection by repeated infection with ticks, suggesting that tick-secreted proteins play a role in immunity against challenge infection [43],[63]. An *H. longicornis* cDNA expression library was immunoscreened using antibodies to *H. longicornis* generated in rabbits by repeated infection of adult ticks [31]. We thereby found a cDNA encoding the putative longipain among several clones that were immunoreactive with the *H. longicornis* immunized rabbit serum. The nucleotide sequences of the cDNAs were determined by the Sanger dideoxy chain termination method, using a PRISMTM Ready Dye Terminator Cycle Sequencing Kit (Perkin-Elmer, http://las.perkinelmer.com). GENETYX-WINTM sequence analysis software and the BLAST network server of the National Center for Biotechnology Information (NCBI) were used to analyze the nucleotides and deduce the amino acid sequences for determining homologies with previously reported sequences in GenBank. The SignalP 3.0 program (Center for Biological Sequence Analysis Biocentrum-DTU, http://www.cbs.dtu.dk/services/SignalP) was used for the prediction of the cleavage site for the signal peptide [64]. Potential N-glycosylation sites were analyzed with the ScanProsite program (Alexandre Gattiker and the Swiss Institute of Bioinformatics) [65]. The amino acid sequence of longipain was aligned with the sequences of known cysteine protease family members using the alignment program ClustalW (http://www.ddbj.nig.ac.jp./E-mail/clustalw-j.html).

### Production of an antibody against recombinant longipain produced in *E. coli*

A specific antibody against longipain was generated in mice (Japan SLC, http://www.jslc.co.jp) immunized with *E. coli*-expressed longipain. The entire coding region of longipain except the signal sequence was subcloned into a plasmid expression vector, pTrcHisB (Invitrogen, http://www.invitrogen.com), as described [29]. The plasmid was transformed into *E. coli* strain TOP10F' (Invitrogen) and the purification process was monitored by SDS-PAGE using a T7 Taq® monoclonal antibody (Novagen, http://www.merckbiosciences.com). The recombinant protein was purified using AKTA equipped with a HiTrap chlelating HP column (GE Healthcare, https://www1.gelifesciences.com). Mice were immunized with 50 µg of the longipain using TiterMax Gold (CytRx, http://www.titermax.com) and boosted two more times as described previously [66]. Serum was prepared from blood collected 2 weeks after the final immunization.

### Expression of the soluble longipain in *Pichia pastoris*

The partial coding region of longipain was amplified by PCR using a sense primer, *longipain* PICZC 5′/*Eco*RI (CC**G AAT TC** T AAT GTC TGA CCG CTA TTT GGT TCC CGT CGA CAT G), which contains an *Eco*RI site (shown in bold) and an antisense primer, *longipain* 3′/*Xho*I (CC**G** **AGC** **TC**G AGA TAT CTA GGT ATT CCA GCT ACA AC), which contains an *Xho*I site (shown in bold) and a yeast initiation consensus sequence (underlined). Competent yeast cells of strain GS115 (Mut+, His-) were prepared according to the protocol of the EasySelect *Pichia* Expression kit (Invitrogen) and transformed with pPICZC-*longipain*. Mut+ transformants were detected after growth on agar minimal methanol+histidine plates (1.34% yeast nitrogen base, 4×10−5% biotin, 0.5% methanol, 1.5% agar) at 30°C for 2 days and subsequently cultured on agar minimal dextrose+histidine plates. For large scale expression of longipain, 50 ml of minimal glycerol+histidine was inoculated with transformed yeast cells and the cells were grown for 2 days on a rotary shaker. Expression was induced by exchanging the medium for 250 ml of minimal methanol+histidine medium and shaking for 24 h at 30°C. Yeast cells were collected by centrifugation at 4°C, 1,600 *g* and resuspended in an equal volume of breaking buffer (50 mM sodium phosphate, pH 7.4, 1 mM EDTA, 5% glycerol) containing 1 mM PMSF (phenylmethylsulfonyl fluoride), and homogenized with an equal volume of acid-washed glass beads (0.5 mm), and the supernatant was obtained by centrifugation at 4°C, 26,400 *g*. The longipain was purified with the use of AKTA equipped with a HiTrap chlelating HP column and was dialyzed in an Slide-A-Lyzer Dialysis Cassette (Pierce, http://www.piercenet.com)

### One- and two-dimensional immunoblot analysis and tick immunohistochemistry

One- and two-dimensional immunoblotting of *H. longicornis* was performed as described previously [29]. Adult female tick protein extract was subjected to one- (SDS-PAGE) or two-dimensional (IEF/SDS-PAGE) electrophoresis, and the proteins were transferred onto a nitrocellulose membrane, and then incubated with mouse anti-longipain antibody (diluted 1∶400). Tick immunohistochemistry was performed with mouse anti-longipain antibody as described previously [66]. The sections on glass slides were incubated with mouse anti-longipain (1∶200). After color development, the sections were observed under a microscope (Axiophot; Carl Zeiss, http://www.zeiss.com).

### Immunoelectron microscopy

The tick midgut was processed as described previously [67]. Thin sections (approximately 80 nm thick) were cut on a Leica UCT ultramicrotome and were mounted on glow-discharged nickel grids and stored on 2% gelatin until labeled. Immunolabeling was performed using mouse anti-longipain (diluted 1∶500) with anti-mouse IgG 12-nm colloidal gold-conjugated secondary antibody. Samples were stained with uranyl acetate and lead citrate and then examined with a Hitachi H-7500 electron microscope.

### Immunofluorescence microscopy

Tick immunofluorescence analysis was performed as described [31]. Bound mouse anti-longipain was detected using anti-mouse IgG Alexa 488 (Invitrogen). The sections were mounted in Vectashield ® (Vector) with 4′,6-diamino-2-phenylindole (DAPI) and photographed with a fluorescence microscope (Leica, http://www.leica-microsystems.com) using appropriate filter sets. Images were collected by using Leica FW4000 software.

### Recovery of lumen contents

The midguts of 72-h-fed female adults were dissected under stereomicroscopic examination and transferred to a tube on ice. Midguts were opened with a needle to release the lumen contents and agitated gently in 50 µl of phosphate buffered saline containing a cocktail of protease inhibitors (Roche, http://www.rocheusa.com). A tube containing the opened midgut and lumen contents was centrifuged at 4°C, 1,600 *g*. The supernatant was collected and used to perform immunoblot analysis for detecting endogenous longipain.

### Enzyme assays

Assays of the activity of the longipain expressed in yeast were performed with fluorogenic substrates (Peptide Institute Inc., http://www.peptide.co.jp) in a final volume of 100 µl containing 25 mM citric acid sodium phosphate and 5 mM dithiothreitol (DTT) [68],[69]. The hydrolysis of Z-Arg-Arg-MCA as a substrate for cathepsin B, Z-Phe-Arg-MCA as a substrate for cathepsin B/L and Suc-Leu-Leu-Val-Tyr-MCA as a substrate for chymotrypsin was measured (Table 1). Each concentration was tested in triplicate. *K_m_* and *k*_cat_ values were determined by fitting initial rate data obtained using multiple substrate concentrations to the Michaelis-Menten equation.

Fluorogenic substrate assays were done with 50 µg/ml of enzyme. Fluorogenic assays were monitored by fluorescence spectrophotometry at 380 nm excitation and 460 nm emission (TECAN, http://www.tecan.com). The optimum pH for the enzyme activity was determined using citrate-sodium phosphate buffer with a pH range of 2.5–8.5 and the optimum temperature was determined using citrate-sodium phosphate buffer with a pH of 5 or 8. The ability of protease inhibitors to inhibit longipain activity against each substrate was also investigated (Table 2).

**Table 2:** Inhibition of longipain by various proteinase inhibitors

Inhibitor	Concentration (mM)	Inhibition (%) relative to control	
		Z-R-R-MCA	Z-F-R-MCA
E-64	0.01	10.0±0.1	84.0±0.2
Antipain	0.05	0	0
Leupeptin	0.10	0	0
PepstatinA	0.10	0	4.1±0.01
PMSF	10.0	20.1±0.1	0

The purified longipain was incubated with fluorogenic substrates in the presence of the protease inhibitors at the indicated concentration. The inhibitory effect is indicated as the percentage of the maximum hydrolytic activity of longipain for each substrate. Data shown are the mean±S.D. (n = 3).

### Preparation of rabbit erythrocyte membranes and assay of hydrolysis by longipain

Haemoglobin-free rabbit erythrocyte membranes were prepared as described below [70],[71]. Two milliliters of freshly drawn rabbit blood were centrifuged at 800 *g*, 4°C and the supernatant was removed. The pellet of erythrocytes was washed with PBS three times and erythrocyte ghosts were prepared by hypotonic lysis in 1 mM phosphate buffer (PB), pH 7.0. Erythrocyte ghosts were washed in 1 mM PB several times to remove internal contents and collected by centrifugation at 12,000 *g*, 4°C, and stored in 1 ml of PBS at 4°C. The membrane stock suspension was diluted 1∶100 in PBS and 15-µl aliquots were incubated with longipain in a final volume of 100 µl containing 25 mM citric acid/sodium phosphate and 5 mM DTT for 6 h at 37°C. The reaction mixtures were subjected to SDS-PAGE and immunoblot analysis using rabbit anti-human spectrin serum (Sigma, http://www.sigmaaldrich.com) at a dilution of 1∶400.

### *Babesia in vitro* culture

*B. equi* merozoites were grown in horse erythrocytes *in vitro* as previously described [62] and incubated in the presence of longipain at different concentrations. Parasitemia was assessed daily by microscopic observation of Giemsa-stained blood smears.

### Detection of biotin-labeled longipain

Longipain was labeled with biotin (Biotin labeling kit-NH_2_, Dojindo, http://www.dojindo.co.jp) according to the manufacturer's protocol. The labeled longipain was added to the culture medium, and the cells were washed several times with PBS. The cells were smeared on slide glasses and fixed in methanol. Biotin-labeled longipain was detected using Alexa 594 streptavidin at a dilution of 1∶500 (Invitrogen). The samples were mounted in Vectashield with DAPI and photographed with a camera-equipped fluorescence microscope as described above.

### RNA interference

The RNAi procedure in ticks was carried out using dsRNA as described previously [72],[73]. The coding sequence of mature longipain was cloned into pBluescript II SK+ plasmid and the inserted sequence was amplified by PCR using the oligonucleotides T7 (5′-GTAATACGACTCACTATAGGGC-3′) and CMo422 primers (5′-GCGTAATACGACTCACTATAGGGAACAAAAGCTGGAGCT-3′) to attach T7 promoter recognition sites at both the 5′ and 3′ ends. The MBP gene was cloned into pBluescript II SK+ plasmid to generate control dsRNA [74]. The inserted sequence was amplified by PCR using a forward primer (5′-TTATGAAAATAAAAACAGGTGCA-3′) and a reverse primer (5′-CTTGTCCTGGAACGCTTTGTC-3′). The PCR products were purified using a gel extraction kit (QIAGEN, http://www.qiagen.com). dsRNA complementary to the DNA insert was synthesized by *in vitro* transcription using T7 RNA polymerase (Promega, http://www.promega.com) according to the manufacturer's protocol. Two micrograms of dsDNA were used as a template and 50–100 µg of dsRNA were synthesized. One microgram of *longipain* dsRNA in 0.5 µl of PBS was injected from the fourth coxae into the haemocoel of unfed adult *H. longicornis* females fixed on a glass slide with adhesive tape. The injections were carried out by using 50-µl microcapillaries (MICROCAP®, Drummond Scientific, http://www.drummondsci.com) drawn to fine-point needles by heating. The needles were connected to an air compressor. Control ticks were injected with 0.5 µl of PBS alone or with 1 µg of *MBP* dsRNA in 0.5 µl of PBS. The ticks were allowed to rest for 1 day at 25°C. No mortality resulted from the injection alone, as both control and *longipain* dsRNA-treated ticks survived after injection while being kept in an incubator prior to placement on the host.

### Infestation of RNAi-treated ticks on a dog that was infected with *B. gibsoni*

The *longipain* dsRNA-injected ticks were placed on the ears of an 8-month-old female beagle (Oriental Bio service, http://www.oyc.co.jp) that was infected with the dog *Babesia* parasite *B. gibsoni*. During attachment, the dog maintained 12% intra-erythrocytic parasitemia in the peripheral blood. The pattern of the control ticks injected with buffer alone was comparable to that of the uninjected ticks used simultaneously to infect the same host. On day 6, ticks were recovered from the dog. The individual organs of ticks were dissected after removal of the midgut contents under a microscope. To verify the gene silencing by *longipain* dsRNA, RT-PCR was performed as described previously [75]. Total mRNA was isolated using a QuickPrep™ Micro mRNA Purification Kit (GE Healthcare) as described in the supplier's protocol. cDNA was then synthesized from 30 µg of mRNA using an RNA PCR Kit (AVM) Ver.3.0 (Takara) following the manufacturer's instructions. PCR was performed using *longipain*-specific oligonucleotides and oligonucleotides specific for *H. longicornis* with 500 ng of cDNA as template in a final volume of 50 µl. PCR products were resolved by 1.8 % agarose gel electrophoresis.

### Detection of *B.gibsoni* and endogenous longipain

Tick sections were prepared as described above. Samples were immunolabeled with mouse anti-*B. gibsoni* (1∶250) and rabbit anti-longipain (1∶150). Bound antibodies were detected using Alexa Fluor 488 goat anti-mouse IgG(H+L) (1∶500) and Alexa Fluor 594 goat anti-rabbit IgG(H+L) (1∶250). The sections were mounted with DAPI and photographed with a fluorescence microscope as described above.

### Real-time PCR assay for quantifying *B. gibsoni* infection

The prevalence and intensity of *B. gibsoni* infection in the dissected organs were evaluated using a real-time quantitative PCR assay. Initially we standardized the PCR protocol using *B. gibsoni* P18 gene-specific primers (D3:5′-TCCGTTCCCACAACACCAGC-3′, D4:5′-TCCTCCTCATCATCCTCATTCG-3′) and purified *B. gibsoni* genomic DNA. *B. gibsoni* P18 which encodes a major surface protein, is a well-known gene that has been demonstrated to be useful as a diagnostic tool for *B. gibsoni* infection [24]). The PCR reaction was performed using a LightCycler 1.5 (Roche) and DNA master SYBR Green I (Roche) with 4 mM MgCl_2_. Standard curves used to quantify relative gene concentrations were made from tenfold serial dilutions of the *B. gibsoni* parasites (genomic DNA) with the following incubation conditions: 95°C×600 s denaturing step, 45 cycles of 95°C×15 s, and 55°C×10 s, and 72°C×15 s, using the Fit Point Method of Light Cycle Software 3.5.3. This protocol resulted in highly specific amplification, with no amplification of dog, tick or a range of other *B. gibsoni* DNAs. Evaluation of the number of *B. gibsoni* using DNA extracted from excised organs was performed according to the established PCR protocol. DNA extraction and determinations of the concentration were performed as described.

### Accession number

Sequence data reported in this manuscript are available from GenBank (http://www.ncbi.nlm. nih.gov/Genbank) under accession number AB255051.
